# Supplementary material for: Retrofitting metal-organic frameworks
Source: Nat Commun. 2019 Oct 29;10:4921. doi: 10.1038/s41467-019-12876-1 (PMC6820732; doi:10.1038/s41467-019-12876-1)
Supplement: Supplementary file 1 — Supplementary Information [file 41467_2019_12876_MOESM1_ESM.pdf]

Supplementary Information

Retrofitting Metal-Organic Frameworks

Schneider et al.

Table of content:

Supplementary Note 1: Definition of the host-guest system 3
Structure of the MOF system 3
Structure of the cross linker 3
Geometric relation between MOF and CL 4
Supplementary Note 2: Input Parameters for RetroFit 7
Structural parameters of the MOF system 7
Structural parameters of the cross linker 7
Single point DFT calculations 7
Supplementary Note 3: Algorithm of the RetroFit program 10
Supplementary Note 4: Restrictions and limitations of RetroFit 13
Supplementary Note 5: Results of the DFT single point calculations 14
Supplementary Note 6: Interpolation error 16
Supplementary Note 7: Results of the RetroFit algorithm 18
Supplementary Note 8: Results from RetroFit for NOTT-100 and NOTT-101 21
Supplementary Note 9: Powder X-ray diffraction 23
Supplementary Note 10: Fourier transform infrared spectroscopy 26
Supplementary Note 11: Scanning electron microscopy 28
Supplementary Note 12: Electrical conductivity measurements 30
Supplementary References 32

Supplementary Note 1: Definition of the host-guest system

The user of RetroFit has to identify the metal-OMS vector of the MOF as well as the vector of the donor groups of the cross linker (CL) to determine the angles γ and α as described in the manuscript. In the following, this is described for the example of a Cu paddlewheel MOF and a nitrile CL. However, this approach can universally be applied to other MOFs and CLs.

Structure of the MOF system

The relative position of two open metal sites in a Cu paddlewheel MOF can be described by the distance between the two Cu atoms (Cu_2 and Cu_4) R_{CuCu} that are available for coordination and the angle of the CuCu vectors of the two paddle wheels γ or by the Cu_1-Cu_2-Cu_4 angle (see Supplementary Figure 1), which are dependent via

$$\gamma = 180° - 2 \cdot (180° - \sphericalangle(Cu_1 - Cu_2 - Cu_4)) \qquad \text{(eq. 1)}$$

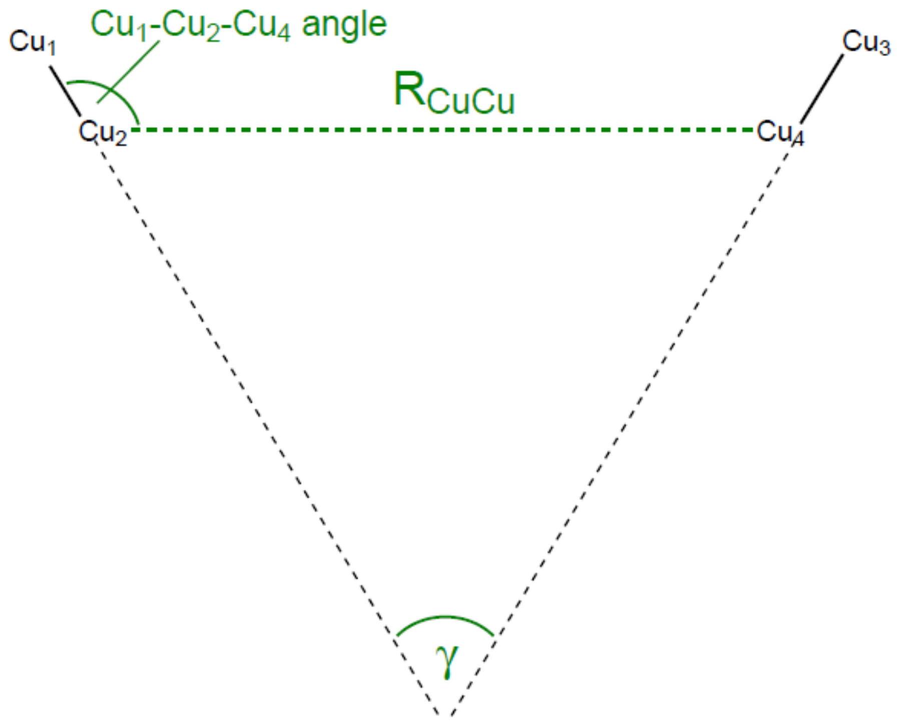

Supplementary Figure 1: Geometric description of the MOF.

Structure of the cross linker

The CL can be described by the distance of the N atoms of the two coordinating nitrile groups R_{NN} and the angle of the two CN vectors α (see Supplementary Figure 2).

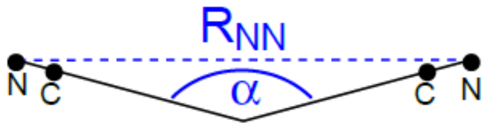

Supplementary Figure 2: Geometric description of the cross linker.

Geometric relation between MOF and CL

The orientation of a CL symmetrically bridging two paddlewheels can be described by the distance between a coordinating nitrile group and the Cu site R_{CuN} and the two angles δ and θ (see Supplementary Figure 3).

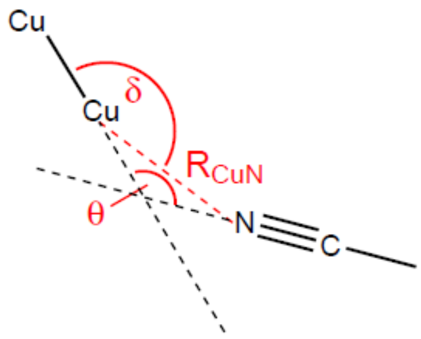

Supplementary Figure 3: Geometric description of the relative orientation of the MOF and the cross linker.

The parameters defining the three subsystems are summarized in Supplementary Table 1.

Supplementary Table 1: Summary of the subsystems that describe the host-guest complex and their respective geometric parameters.

**System**	**Parameters**
Cu paddlewheel MOF	R_{CuCu} and γ<br>or<br>R_{CuCu}, and $\sphericalangle(Cu_1 - Cu_2 - Cu_4)$
Cross linker	R_{NN} and α
Host-Guest	R_{CuN}, δ and θ

Combining the geometric relations from Supplementary Figure 1 - Supplementary Figure 3 defines the entire host-guest complex with seven parameters (see Supplementary Table 1), as illustrated in Supplementary Figure 4.

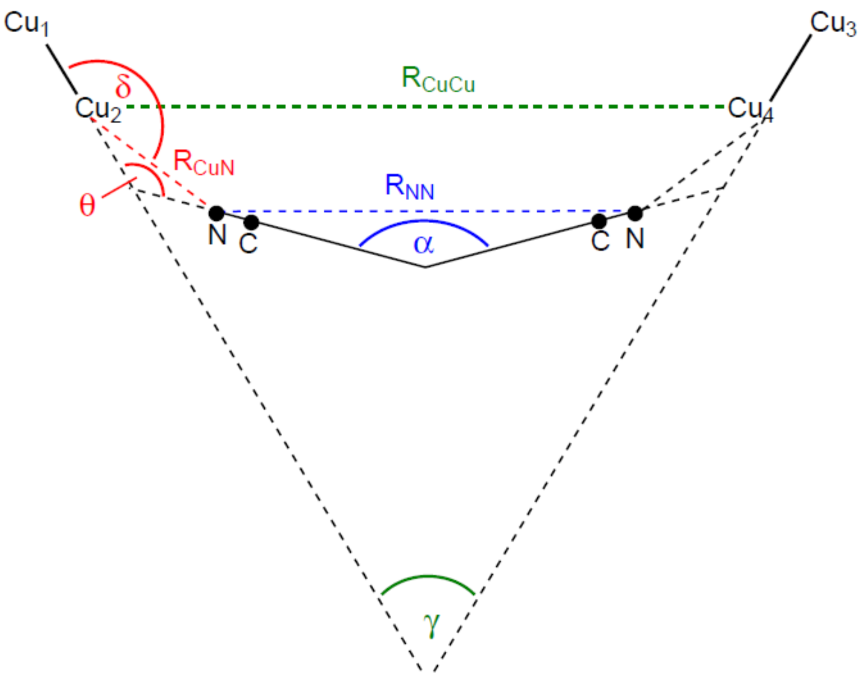

Supplementary Figure 4: 2D representation of the geometric relations defining the host-guest system.

In the following, it is shown that α, θ and γ are interdependent. Therefore, the auxiliary angle κ, which is the angle in the Cu_2-Cu_4-γ triangle, is defined:

$$\kappa = 90° - \frac{\gamma}{2} \qquad \text{(eq. 2)}$$

The angle between N-Cu_2-Cu_4, which is the auxiliary angle ε, is therefore:

$$\varepsilon = \kappa - (180° - \delta) = \delta - \frac{\gamma}{2} - 90° \qquad \text{(eq. 3)}$$

Now, a right-angled triangle, spanned by the distances R_{CuN} and ½(R_{CuCu} - R_{NN}) (including angle ε), can be defined (see Supplementary Figure 5).

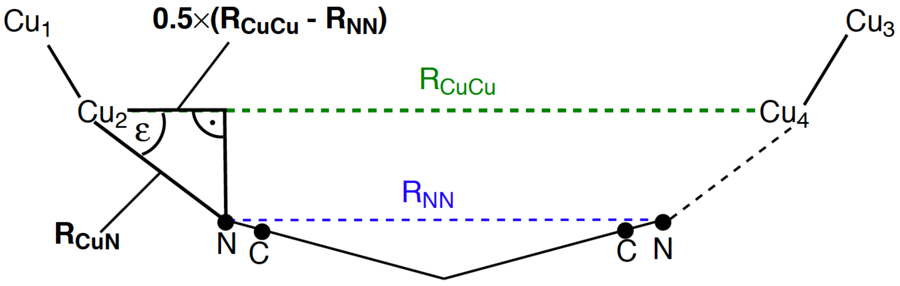

Supplementary Figure 5: Right-angled triangle defined by R_{CuN}, ½(R_{CuCu} - R_{NN}) and ε.

Now, the following cosine relation can be expressed:

$$\cos(\varepsilon) = \cos\left(\delta - \frac{\gamma}{2} - 90°\right) = \sin\left(\delta - \frac{\gamma}{2}\right) = \frac{1}{2}\frac{R_{CuCu} - R_{NN}}{R_{CuN}} \qquad \text{(eq. 4)}$$

In addition, a second triangle can be defined (see Supplementary Figure 6). In this triangle, $\frac{\gamma}{2}$, $180° - \theta$ and $180° - \frac{\alpha}{2}$ have an angle sum of 180°:

$$180° = 180° - \theta + \frac{\gamma}{2} + 180° - \frac{\alpha}{2} \qquad \text{(eq. 5)}$$

This equation can be rearranged to:

$$\alpha = 360° + \gamma - 2\theta \qquad \text{(eq. 6)}$$

Consequently, the entire host-guest system can be described by six variables, i.e. R_{CuCu}, R_{NN}, R_{CuN}, α, γ, and δ, or R_{CuCu}, R_{NN}, R_{CuN}, α, θ, and δ, or R_{CuCu}, R_{NN}, R_{CuN}, α, γ, and θ.

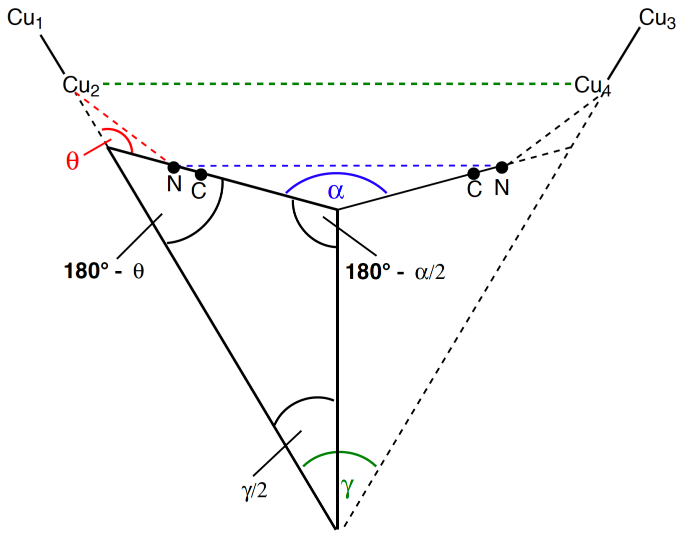

Supplementary Figure 6: Right-angled triangle defined by $\frac{\gamma}{2}$, $180° - \theta$ and $180° - \frac{\alpha}{2}$.

In the retrofitting tool, the input parameters of a molecule (α and R_{NN}) and of the MOF-system (R_{CuCu} and γ) are used. As the description of the whole system requires 6 variables, a screening of the 2 missing variables (R_{CuN} and δ) is required in order to find the optimal position on a MIP (see below).

Supplementary Note 2: Input Parameters for RetroFit

Structural parameters of the MOF system

The geometric information of the MOF system (R_{CuCu} and γ) is extracted from the crystal structure (cif-file) of the MOF and is a manual input. Please refer to the How-To for further information.

Structural parameters of the cross linker

All molecule geometries were optimized with the Gaussian09 program package.[1] Optimization was performed with DFT with a B3LYP hybrid functional and a 6-31G basis set.[2, 3] For the optimization the *tight* convergence criterion was used and the Hessian was recalculated after each optimization step (keyword 'calcall'). All molecules were symmetry restricted during the optimization process (see Supplementary Figure 7). The resulting Gaussian output file was converted to the xyz-format using Open Babel (version 2.3.2) and then imported into the retrofit tool using the Atomic Simulation Environment (ASE) (version 3.16.0) to compute R_{NN} and α.

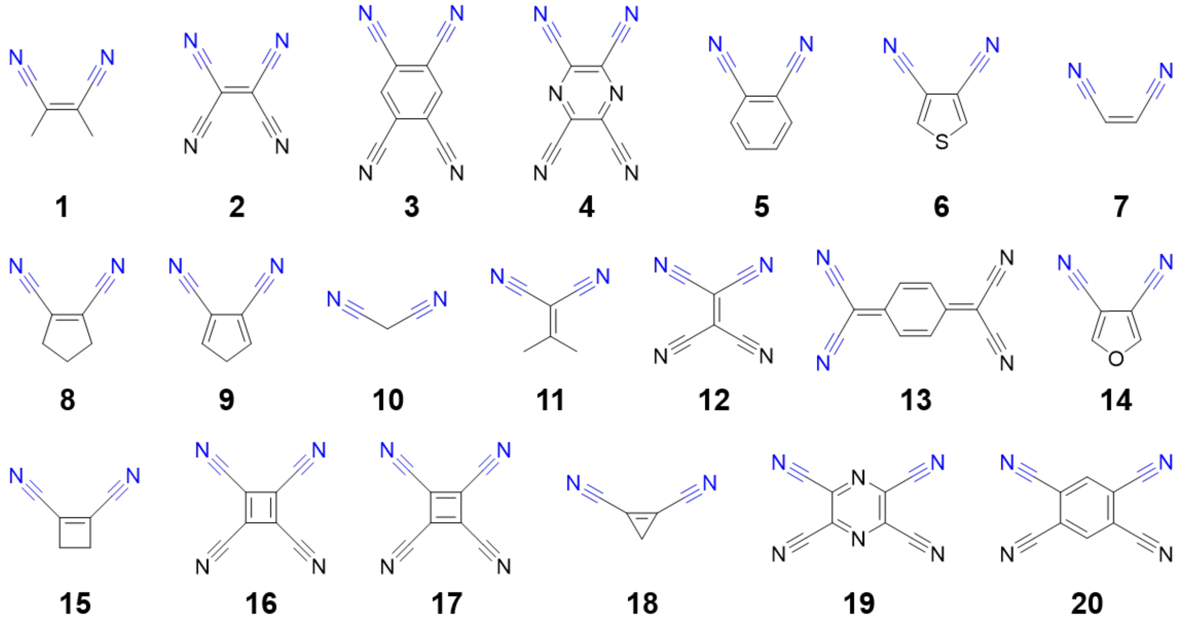

Supplementary Figure 7: Library of dicyano-CLs used in this work. CLs 2, 3, 4, 12, 13, 16, 17, 19, and 29 were restricted to D_{2h} symmetry while CLs 1, 5-11, 14, 15, and 18 were restricted to C_{2v} symmetry during the optimization with Gaussian.

Single point DFT calculations

To access energies in the host-guest system, a simple model was chosen and transferred to the real system. Precisely, we optimized a Cu(II) formate paddlewheel and an acetonitrile molecule, respectively, and then arranged the two entities that the nitrile group points towards the OMS of the paddlewheel. By varying R_{CuN}, δ and θ according to the parameters given in Supplementary Table 2, we obtained an energy of the system for every combination of the three parameters, which allows the generation of a model interaction potential (MIP). Two screenings were performed: one with the acetonitrile in the Cu-O plane and another one with the acetonitrile in the plane of the O-Cu-O angle bisector. Both screenings yielded similar energy values and therefore

we proceeded with the values for the in-plane scan. The configuration with the lowest energy is defined as 0 kcal/mol and all energies are given as energy differences ∆E.

The single point calculations were done on a DFT level of theory using the TURBOMOLE (V7.1) software package.[4] The hybrid functional B3LYP[3, 5] was used with a TZVPP basis set[6] and a fine ‘m5’ grid[7] for all elements. The multipole-accelerated[8] resolution of the identity approximation[9, 10] was used for performance reasons. Grimmes D3[11] was employed to properly account for dispersive interactions. The SCF convergence criterion was set to 10^{-6} Hartree.

The spin-state of the Cu^{2+} dimer with a total of 18 d-electrons was assigned to be the ferromagnetically coupled triplett state (eight occupied d orbitals and two singly occupied d orbitals with alpha spin). Even though the spin state in a Copper paddlewheel MOF is the open-shell singled state(eight occupied d orbitals and two singly occupied d-orbitals, one with alpha and one with beta spin), the excited state we chose is experimentally only 292.2 cm^{-1},[12] and in a comparable hybrid (B3LYP/cc-pVDZ-PP/UDFT) calculation 372 cm^{-1} above the ground state energy,[13] which at room temperature makes it notably populated. Computing the antiferromagnetically coupled ground state or applying e.g. a multireference treatment however, requres a more involved theoretical description, which is why we chose here to focus on the low lying excited state. This was justified in the aforementioned computational study, where the two states were compared with respect to their energies, geometries and vibrational frequencies/modes and were found to be “nearly indistinguishable”.[13]

Due to the electronic configuration, the calculations were carried out spin-unrestricted.

Supplementary Table 2: Details about the scanning grid for the MIP of the paddlewheel – acetonitrile system.

System	Start	End	Step size
R_{CuN}	1.8 Å	5.0 Å	0.2 Å
δ	90°	180°	5°
θ	90°	270°	5°

Due to symmetry reasons, we can mirror the resulting three-dimensional MIP for displacements of δ and θ >180° according to eq. 7 and Supplementary Figure 8. This way we obtained a MIP in the intervals R_{CuN} = [1.8 Å, 5.0 Å], δ = [90°, 270°], θ = [90°, 270°].

$$E(\mathrm{R_{CuN}}, \delta, \theta) = E(\mathrm{R_{CuN}}, 360-\delta, 360-\theta) \quad \text{(eq. 7)}$$

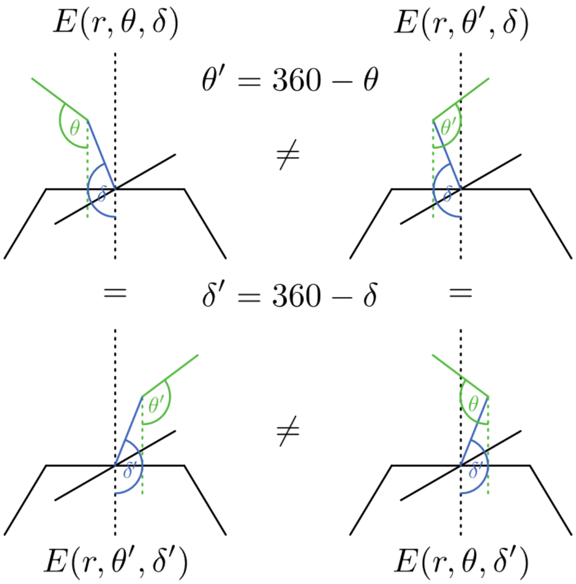

Supplementary Figure 8: Symmetry relations used to mirror the energies obtained from DFT calculations.

Supplementary Note 3: Algorithm of the RetroFit program

The program code is written in the open source programming language Python™ (available at http://www.python.org). First, the data of the paddlewheel MIP (R_{MD}, δ, θ and energy ΔE) is imported and interpolated. As interpolation method, a regular grid interpolator from the Scipy package was used (the class scipy.interpolate.RegularGridInterpolator). This interpolation method uses trilinear interpolation. As method-tag, 'linear' was chosen. The interpolation results in an object, which has as input R_{MD}, δ and θ and returns the respective energy value E (i.e. ΔE) and works within the boundaries of the MIP as defined in Supplementary Table 2, including the mirroring of δ and θ for angles >180°. Second, the geometric information of the MOF system (R_{MM} and $\sphericalangle(M_1 - M_2 - M_4)$, although γ is used as describing angle in rest of the program) and guest molecules (α, R_{DD}) are imported. Note, for the ease of use the $\sphericalangle(M_1 - M_2 - M_4)$ is used as input value as it can be easily obtained from the MOF crystal structure. For non-paddlewheel MOFs this angle is defined by the vector M-M and a virtual vector between one metal center and the direction of its OMS. This data is used to calculate via (eq. 4) and (eq. 6) an energetically optimal triple of R_{MD}, δ and θ. Hereby, θ can be easily calculated from α and γ with (eq. 6). In order to obtain R_{MD} and δ, (eq. 6) is used. As this equation has two unknown variables, it is not straight forward to calculate both quantities simultaneously. Therefore, a set of δ values (δ -testlist) is used to calculate a respective set of R_{MD} values. Subsequently, the δ -testlist, the set of R_{MD} values and θ is used to calculate with the interpolation object a set of energies. The lowest energy value of the set is identified. By doing this, the energetically most favorable coordinates R_{MD}, δ and θ of a given guest molecule with the geometry defined by α and R_{DD} in a MOF system defined by R_{MM} and γ are obtained for the respective input MIP data. A flowchart of the described algorithm is shown in Supplementary Figure 9.

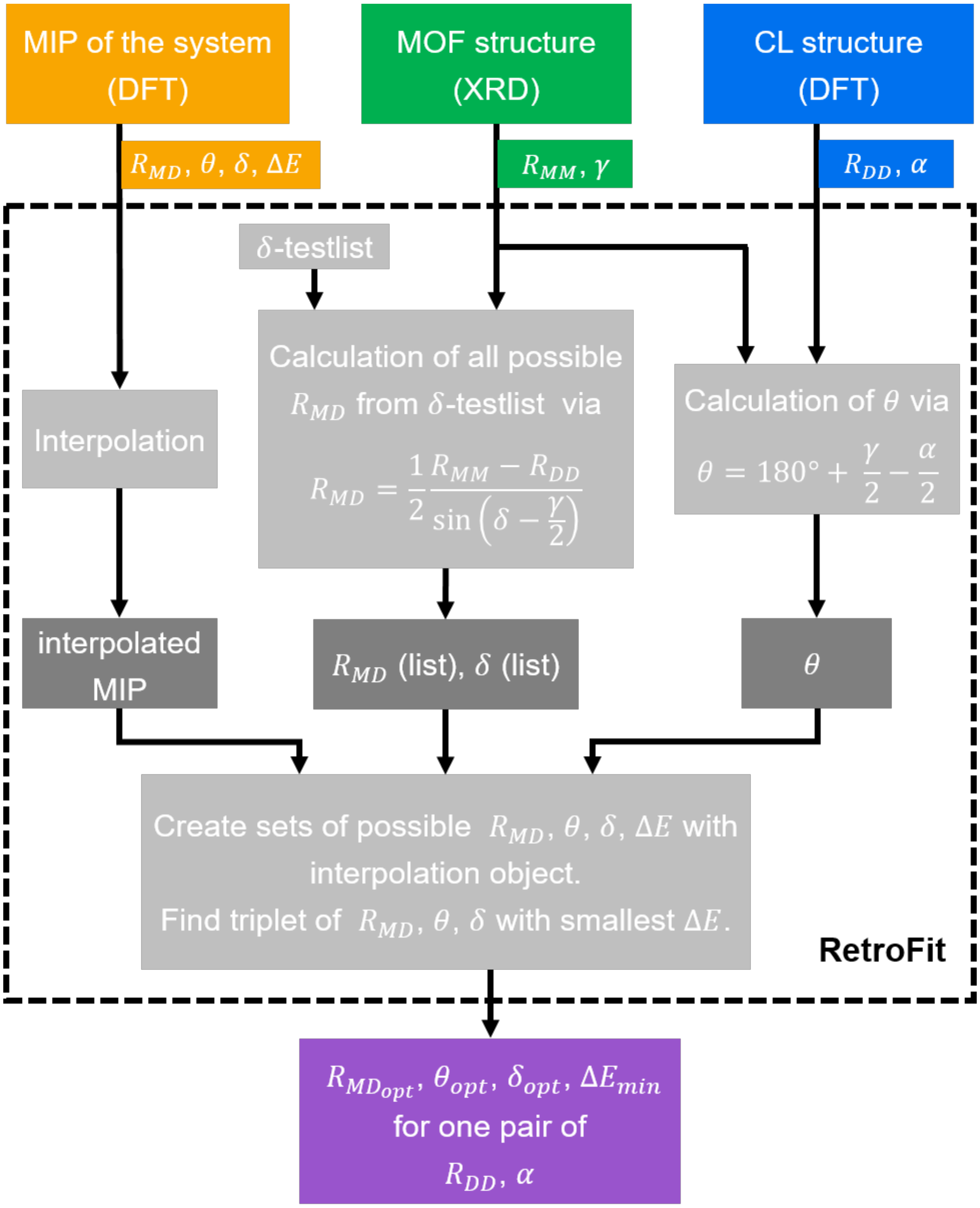

Supplementary Figure 9: General workflow of the RetroFit program with emphasis on the various steps that are performed within the Python-based code. The result of one cycle is a ΔE_{min} value (purple) for a given CL@MOF system. When performed for various CLs for one parent MOF with OMS, CLs can be ranked with respect to their applicability in a retrofit experiment based on their ΔE_{min} values. A how-to guideline is provided as a separate part of the supplementary information.

The execution time of the code depends on the selected step size of the δ-testlist and the step size of the parameters α and R_{NN} used to generate the energy map. Using a step size of 100 for the three parameters, which we found reasonable, the execution time is in the order of a few minutes sing a typical personal computer (Supplementary Figure 10).

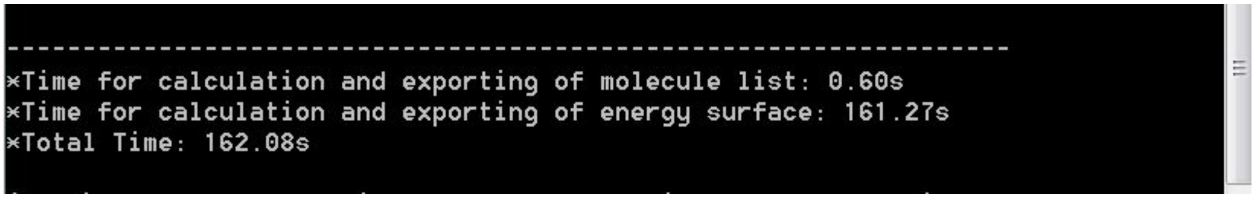

Supplementary Figure 10: Screenshot of the reported runtime of RetroFit. The code was executed on a personal computer (Windows 10) using a step size of 100 for δ, α and R_{NN}.

Supplementary Note 4: Restrictions and limitations of RetroFit

Open metal-site of MOF

In this model, all four Cu atoms are located in one plane and consequently the Cu-OMS vector is also located in that plane. Therefore, this model works for 2-dimensional systems that have a mirror plane, meaning at least a C_{2v} symmetry. For systems in which the Cu-OMS vectors are not in one plane additional assumptions are necessary (see results for the NOTT systems below).

Guest Molecule

All guest molecules are defined by their functional groups, which are in this case nitrile groups. The two nitrile groups (i.e. the C-N vectors) have to be in one plane for the system to be mirror symmetric to each other. Hence, a C_{2v} symmetry of this four-atom system is required, or further assumptions are necessary.

Interaction potential of model system

For every new metal – functional group pair a new MIP has to be calculated, which requires some DFT calculations.

Interpolation

In the retrofit program an interpolation of the MIP data is performed. Hence, interpolated data are only available in the limits of the calculated MIP data (see Supplementary Table 2).

General

As no geometrical optimization is performed by the RetroFit program, all components are considered rigid. Therefore, the framework and the molecule do not change their geometry and bond lengths and angles are always constant in each subsystem. In addition, no dispersion interactions or interactions between two guest molecules are taken into consideration. For a more accurate simulation, the entire system had to be calculated by DFT at significantly higher computational costs.

Supplementary Note 5: Results of the DFT single point calculations

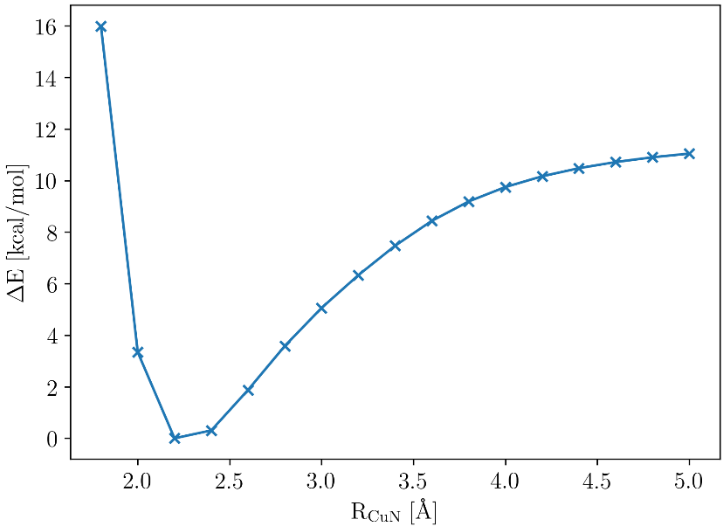

Supplementary Figure 11: Slice of the acetonitrile – Cu paddlewheel MIP with δ = 180° and θ = 180°.

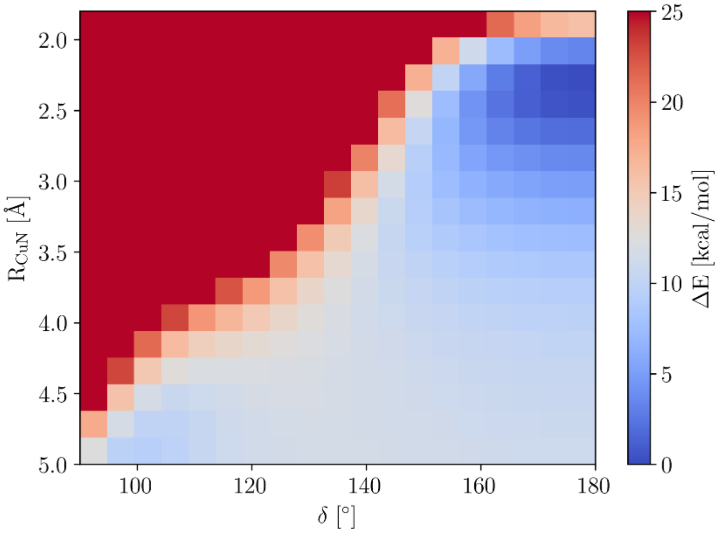

Supplementary Figure 12: Slice of the acetonitrile – Cu paddlewheel MIP with θ = 180°.

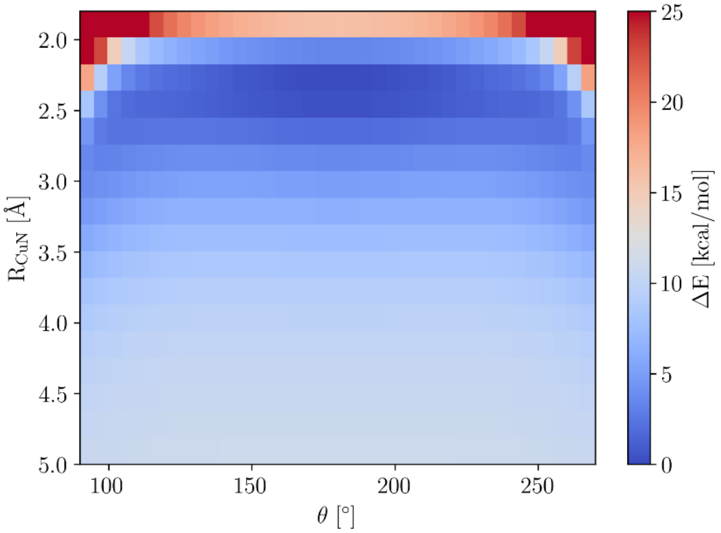

Supplementary Figure 13: Slice of the acetonitrile – Cu paddlewheel MIP with δ = 180°.

Supplementary Note 6: Interpolation error

In order to verify the interpolation of the the MIP, we have checked for error values originating from the interpolation itself. The set of Energies $E(\{\alpha,\theta,\delta\})$ was linearly interpolated to obtain a hypothesis $\hat{E}(\{\alpha,\theta,\delta\})$ for any point inside the set of linearly spaced points $\alpha_i, \theta_i, \delta_i$.

The Error of interpolation at any of these points is given by

$$ERR(\{\alpha,\theta,\delta\}) = E(\{\alpha,\theta,\delta\}) - \hat{E}(\{\alpha,\theta,\delta\}) \quad \text{(eq. 8)}$$

We divide the space into cubelets with dimensions 0.2 Å×5°×5°. For each cubelet, a maximum error is estimated.

In order to find an upper limit for the error in each cubelet, the diagonal of the cubelet is considered (from $\alpha_i, \theta_i, \delta_i$ to $\alpha_{i+1}, \theta_{i+1}, \delta_{i+1}$).

According to the mean value theorem, we can estimate the interpolation error for a linear interpolation of a point x between two adjacent linearly spaced points x_i and x_{i+1} (e.g $x \in [x_i, x_{i+1}]$) in the following way:

$$ERR(x) \leq \max_{x \in [x_i, x_{i+1}]} \left(\frac{(x - x_i)(x - x_{i+1})}{2} \right) \cdot \max_{x' \in [x_i, x_{i+1}]} \left(f^{(2)}(x') \right) \quad \text{(eq. 9)}$$

$f^{(2)}(x')$ is the second derivative for $x \in [x_i, x_{i+1}]$. Since the maximum value of the derivative is not known, we estimate it from the numerically calculated second derivatives at the nearest evaluation points x_i and x_{i+1} and choose the largest value of these two.

As we have a multi-dimensional problem, we estimate the error in each direction along the sides of the cubelet (from $\alpha_i, \theta_i, \delta_i$ to $\alpha_{i+1}, \theta_i, \delta_i$, from $\alpha_i, \theta_i, \delta_i$ to $\alpha_{i+1}, \theta_i, \delta_i$ and from $\alpha_i, \theta_{i+1}, \delta_i$ to $\alpha_i, \theta_i, \delta_{i+1}$) and then estimate the total error in a cubelet as:

$$ERR(\{\alpha,\theta,\delta\}) = \sqrt{{ERR_r}^2 + {ERR_\theta}^2 + {ERR_\delta}^2} \quad \text{(eq. 10)}$$

Supplementary Figure 14 depicts the interpolation errors for the slices R_{CuN} = 2.3, 2.5 and 2.7 Å with insets of all of the evaluated CL molecules. All molecules are in a low-error area and hence our results are inside a reasonable margin of error. The output of RetroFit provides energy penalties with full precision. Based on these considerations, we suggest to give the energy penalties as within the accuracy of three decimal places, as done for our retrofitted test-systems, see Supplementary Note 7.

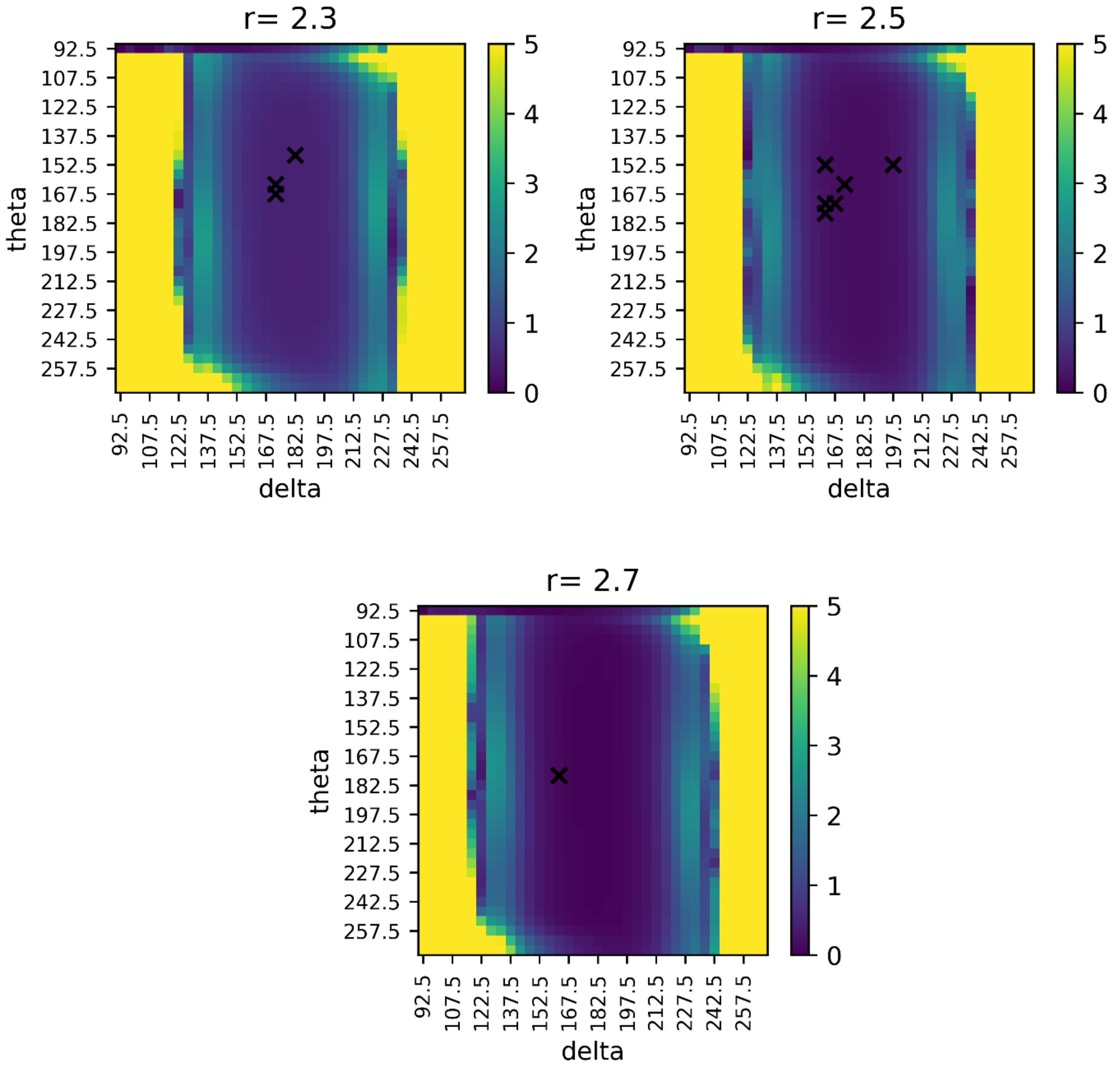

Supplementary Figure 14: Interpolation errors of the interpolated acetonitrile – Cu paddlewheel MIP. For visualization purposes, slices of the four-dimensional MIP are shown at R_{CuN} = 2.3, 2.5, and 2.7 Å. The error increases from dark blue to yellow and the scale is given in kcal / mol. The CLs tested in this study are indicated by a black "x" and are all located in the low-error area.

Supplementary Note 7: Results of the RetroFit algorithm

RetroFit calculates the optimal position of a CL (R_{CuN}, δ, θ) within a given MOF and provides energy deviation ΔE from the ideal configuration. This allows to rank the tested CLs according to their fit, i.e. lowest ΔE values, and represents a guideline for experimentalists. The energies for all tested CLs and MOFs studied in this work are provided in Supplementary Table 3.

Supplementary Table 3: Energy penalties ΔE calculated for the fit of different dicyano-CLs in Cu_3BTC_2, NOTT-100 and NOTT-101 using the RetroFit algorithm.

			ΔE / kcal mol^{-1}				
**CL**	**Guest**	R_{NN} / Å	**Cu_3BTC_2**	**NOTT-100 3,5-pos.**	**NOTT-100 3,3'-pos.**	**NOTT-101 3,5-pos.**	**NOTT-101 3,3"-pos.**
**1**	1,2-dimethyl-1,2-dicyano-ethylene	4.077	4.203	3.835	0.311	4.281	9.881
**2**	TCNE (cis)	4.095	4.175	3.826	0.349	4.252	9.864
**3**	TCNB (ortho)	4.114	4.107	3.746	0.339	4.182	9.842
**4**	tetracyano-pyrazine (ortho)	4.127	4.077	3.705	0.339	4.151	9.828
**5**	1,2-dicyano-benzene	4.134	4.014	3.642	0.318	4.089	9.817
**6**	3,4-dicyano-thiophen	4.333	3.175	2.864	0.606	3.238	9.541
**7**	cis-dicyanoethylene	4.370	2.965	2.646	0.723	3.030	9.469
**8**	1,2-dicyanocyclopentene	4.424	2.847	2.522	0.793	2.908	9.366
**9**	dicyanocyclopentadiene	4.424	2.847	2.522	0.793	2.908	9.366
**10**	malononitrile	4.431	2.317	1.964	1.266	2.395	9.211
**11**	dicyano-2-methyl-propene	4.433	2.305	1.952	1.291	2.383	9.203
**12**	TCNE (geminal)	4.436	2.292	1.940	1.289	2.371	9.197
**13**	TCNQ (geminal)	4.447	2.241	1.889	1.328	2.319	9.170
**14**	3,4-dicyanofuran	4.551	2.330	1.949	1.286	2.405	9.176
**15**	1,2-dicyanocyclobutene	5.016	0.789	0.664	5.299	0.837	8.157
**16**	tetracyanocyclobutene (single bond)	5.082	0.702	0.535	5.482	0.708	8.043

**17**	tetracyanocyclobutene (double bond)	5.132	0.650	0.494	6.794	0.658	7.847
**18**	1,2-dicyanocyclopropene	5.772	0.797	0.870	11.682	0.744	5.919
**19**	tetracyano-pyrazine (meta)	6.761	4.049	4.596	12.097	3.976	2.333
**20**	TCNB (meta)	6.893	4.815	5.472	15.884	4.689	1.882

Categorization of the energy penalties:

In order to evaluate and categorize the output values of RetroFit, i.e. the energy penalty Δ*E*, it is worth looking at the binding energy of the entire retrofitted system (CL@MOF). Considering the two coordination bonds formed between a CL and the MOF, the energy gain E_{Retrofit} can be approximated by (eq. 11).

$$E_{Retrofit} = 2\ (E_{Binding} - \Delta E) \qquad \text{(eq. 11)}$$

From the acetonitrile – Cu paddlewheel MIP at δ = 180° and θ = 180° (Supplementary Figure 11) we can calculate the binding energy E_{Binding} of the ideal configuration as

$$E_{Binding} = E(R_{CuN} = 5.0) - E(R_{CuN} = 2.2) = 11.05\ kcal/mol. \qquad \text{(eq. 12)}$$

A ditopic binding situation, i.e. retrofitting, is favored over a monotopic coordination of only one donor group to the OMS, if

$$E_{Retrofit} > E_{Binding}. \qquad \text{(eq. 13)}$$

From (eq. 11) and (eq. 13) follows that retrofitting of nitrile-CLs in Cu-paddlewheel MOFs is favored over a monotopic coordination if

$$\Delta E < \frac{1}{2} E_{Binding} \approx 5.5\ kcal/mol. \qquad \text{(eq. 14)}$$

For the results in this study (see Supplementary Table 3) we suggest the general categorization shown in Supplementary Table 4.

Supplementary Table 4: Proposed categorization of the RetroFit results.

ΔE / kcal mol^{-1}	Category
0 – 2.5	good fit
2.5 – 5.0	medium fit
> 5.0	bad / no fit

Supplementary Table 5: Comparison of the input parameters and results of the retrofit program and the DFT calculation of TCNQ@Cu_3BTC_2 of reference [14].

Parameter	DFT[14]	RetroFit[§]	RetroFit with perturbation[$]
R_{CuCu} / Å	7.99969	7.9997	7.9997
γ / °	60.0	60.0	60.0
R_{NN} / Å	4.614	4.447	4.614
α / °	132.34	118.42	123.34
R_{CuN} / Å	2.324	2.492	2.397
δ / °	163.08	164.54	165.08
θ / °	143.75	150.79	143.8
ΔE / kcal mol^{-1}	-	2.24	1.59

[§] Values obtained following the regular RetroFit routine. R_{NN} and α for TCNQ were optimized as described above.

[$] Using R_{NN} and α for TCNQ from the DFT optimized structure,[14] i.e. taking into account the distortion of the CL, results in a lower ΔE, showing that RetroFit slightly overestimates ΔE (see Supplementary Note 4: Restrictions and limitations of RetroFit).

Supplementary Note 8: Results from RetroFit for NOTT-100 and NOTT-101

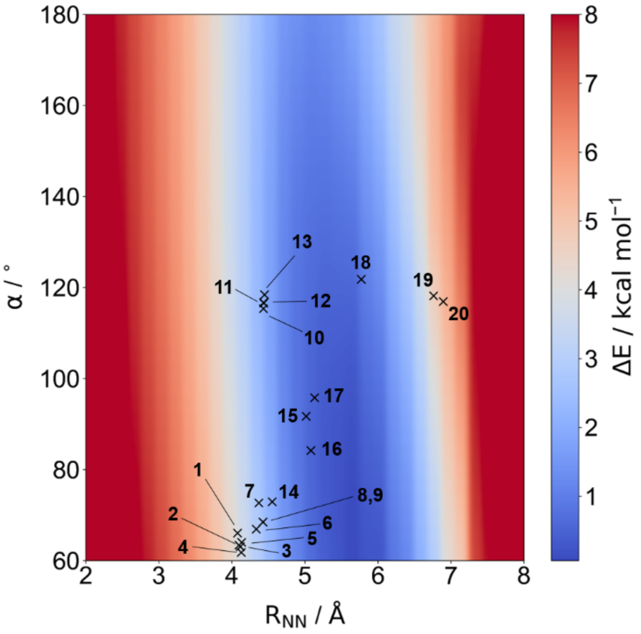

Supplementary Figure 15: RetroFit map for the 3,5-position of NOTT-100 and dinitrile-CLs. The energy penalty ΔE for the CL parameters R_{NN} and α is given as a color code increasing from blue to red. The tested molecules are marked on the map and listed on the right side with increasing ΔE. Energies exceeding the color scale bar are set to 8 kcal/mol for better visualization.

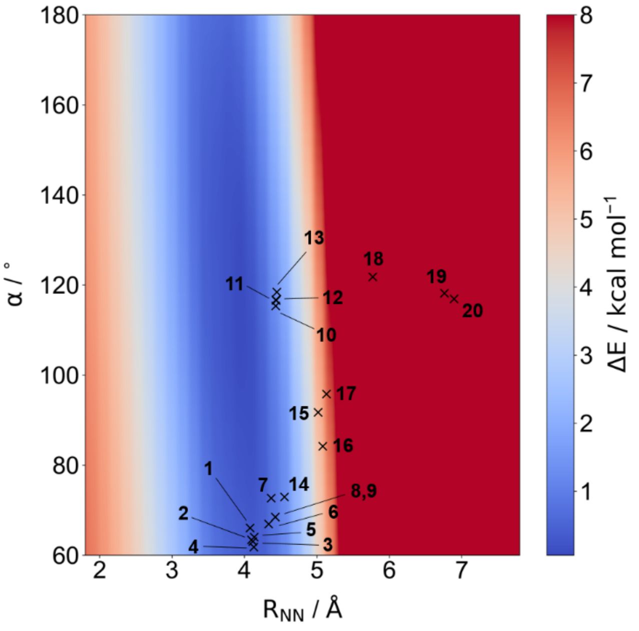

Supplementary Figure 16: RetroFit map for the 3,3'-position of NOTT-100 and dinitrile-CLs. The energy penalty ΔE for the CL parameters R_{NN} and α is given as a color code increasing from blue to red. The tested molecules are marked on the map and listed on the right side with increasing ΔE. Energies exceeding the color scale bar are set to 8 kcal/mol for better visualization.

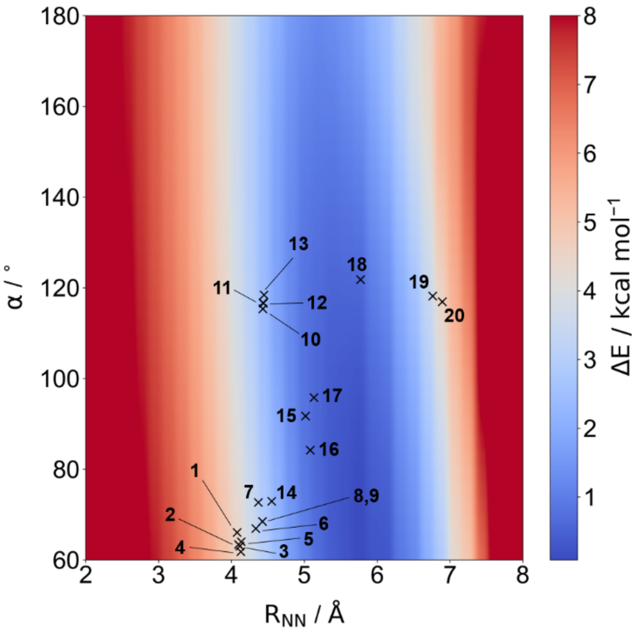

Supplementary Figure 17: RetroFit map for the 3,5-position of NOTT-101 and dinitrile-CLs. The energy penalty Δ*E* for the CL parameters R_{NN} and α is given as a color code increasing from blue to red. The tested molecules are marked on the map and listed on the right side with increasing Δ*E*. Energies exceeding the color scale bar are set to 8 kcal/mol for better visualization.

Supplementary Note 9: Powder X-ray diffraction

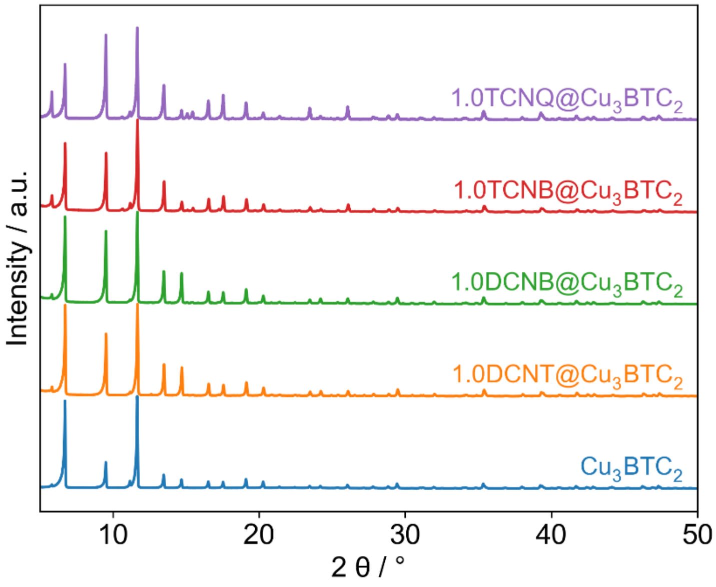

Supplementary Figure 18: Powder X-ray diffractograms of Cu_3BTC_2 (blue) and CL@Cu_3BTC_2 with CL = DCNT (orange), DCNB (green), TCNB (red), TCNQ (purple).

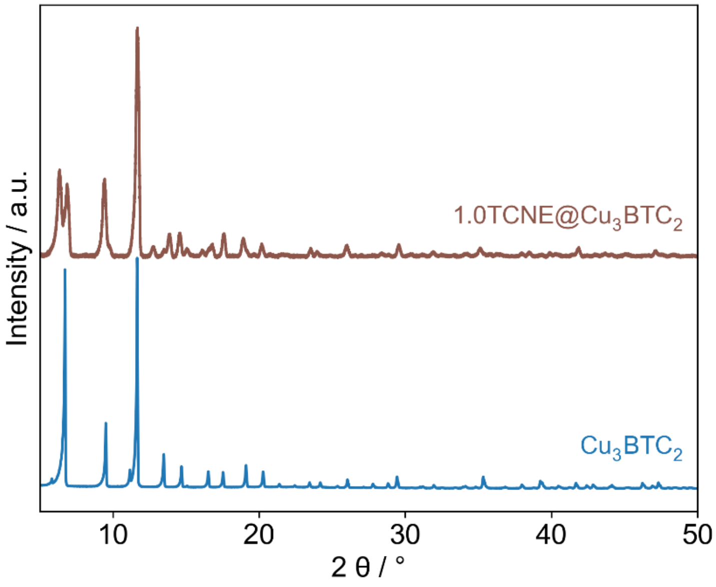

Supplementary Figure 19: Powder X-ray diffractograms of TCNE@Cu_3BTC_2 (brown) and Cu_3BTC_2 (blue).

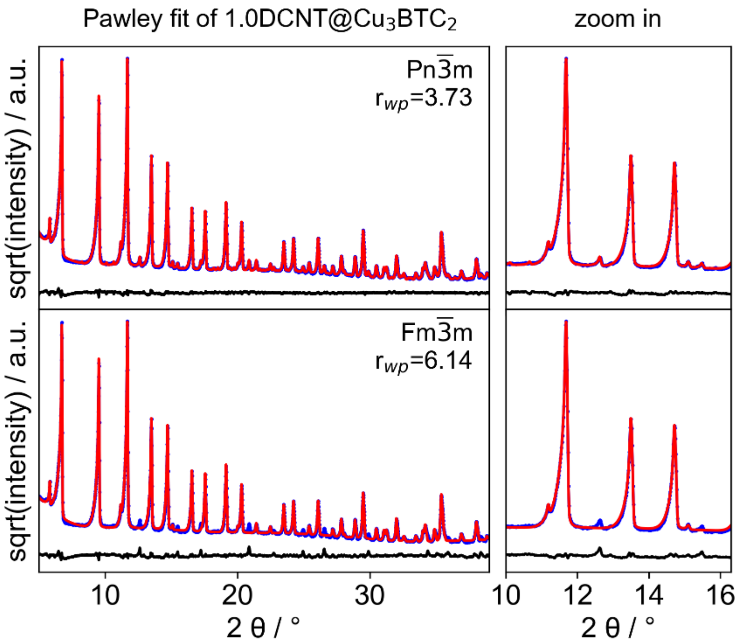

Supplementary Figure 20: Comparison of the Pawley profile fits for PXRD data of DCNT@Cu_3BTC_2 using the crystallographic space groups Fm-3m and Pn-3m.

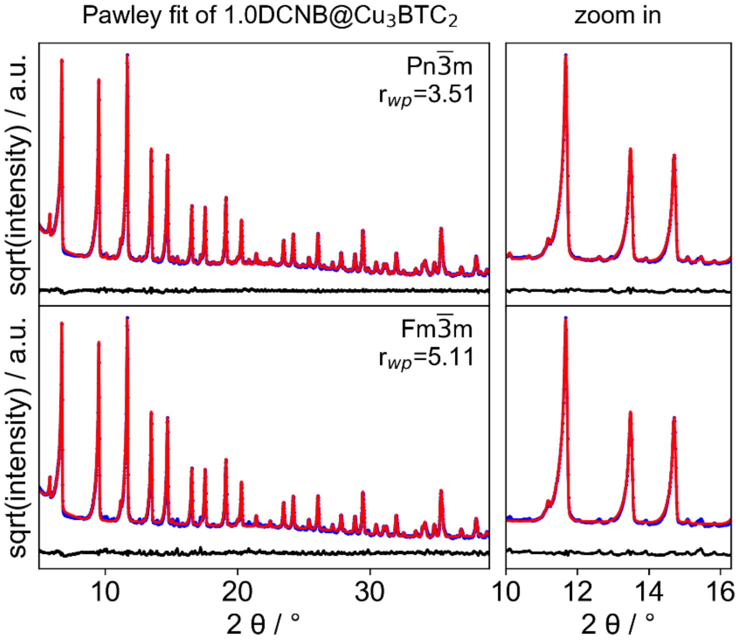

Supplementary Figure 21: Comparison of the Pawley profile fits for PXRD data of DCNB@Cu_3BTC_2 using the crystallographic space groups Fm-3m and Pn-3m.

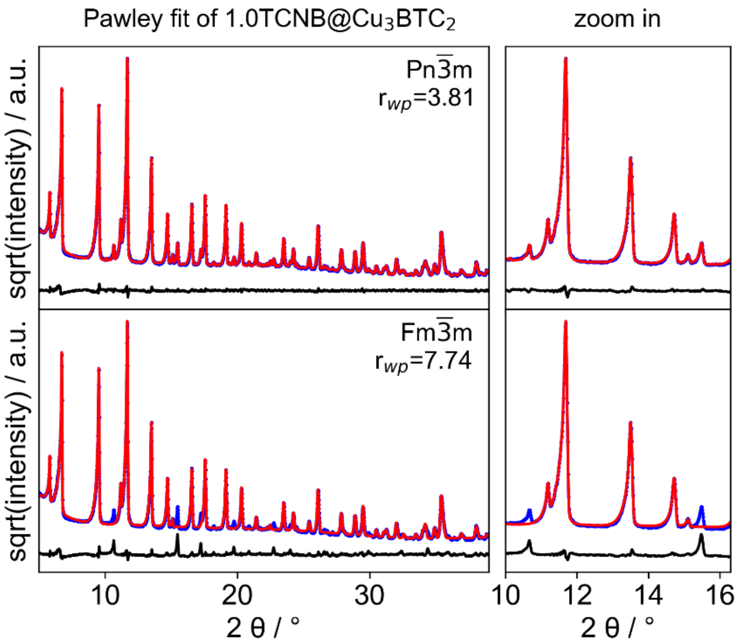

Supplementary Figure 22: Comparison of the Pawley profile fits for PXRD data of TCNB@Cu_3BTC_2 using the crystallographic space groups Fm-3m and Pn-3m.

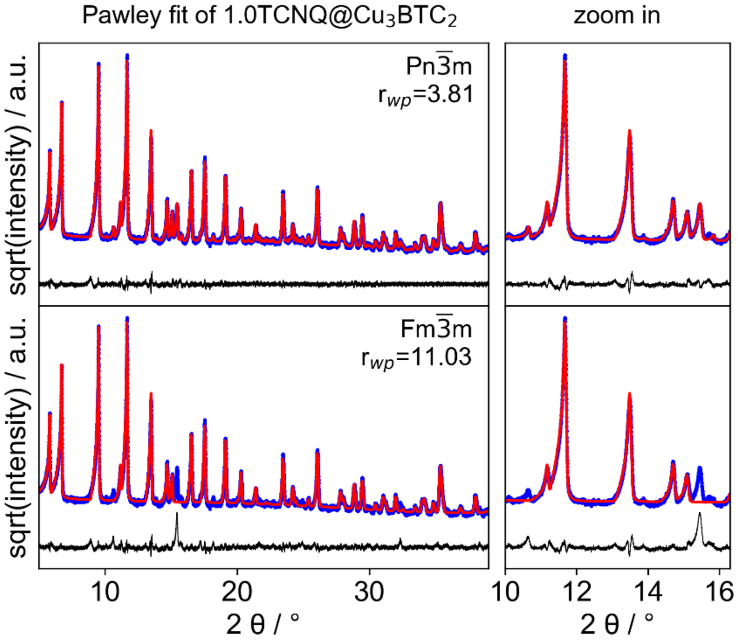

Supplementary Figure 23: Comparison of the Pawley profile fits for PXRD data of TCNQ@Cu_3BTC_2 using the crystallographic space groups Fm-3m and Pn-3m.

Supplementary Note 10: Fourier transform infrared spectroscopy

Fourier transform infrared spectroscopy (FTIR) measurements of powder samples were performed inside an Ar-filled glovebox on an ALPHA FTIR spectrometer (Bruker). The instrument was equipped with a Pt attenuated total reflectance (ATR) unit. 64 scans per measurement were recorded at room temperature in the range of 400–4000 cm^{-1} with a resolution of 2 cm^{-1}.

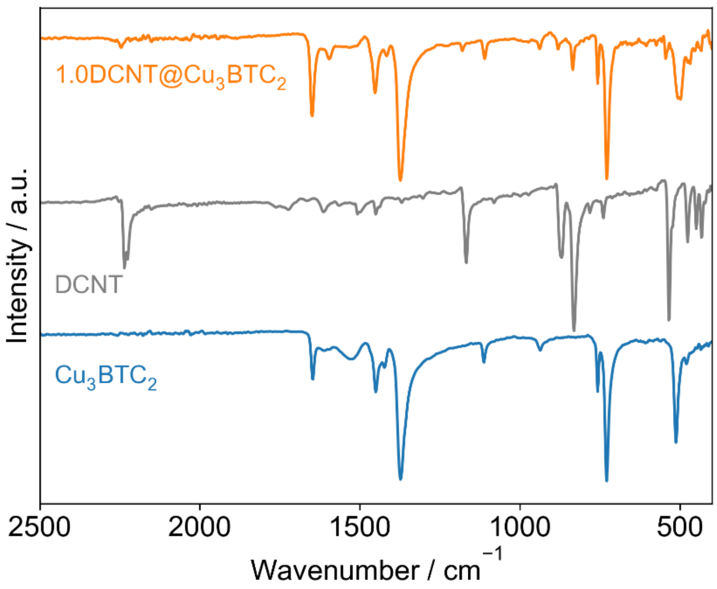

Supplementary Figure 24: IR transmission spectrum of pristine Cu_3BTC_2, DCNT and DCNT@Cu_3BTC_2. The spectra are background corrected and normalized. A vertical offset is applied for visualization.

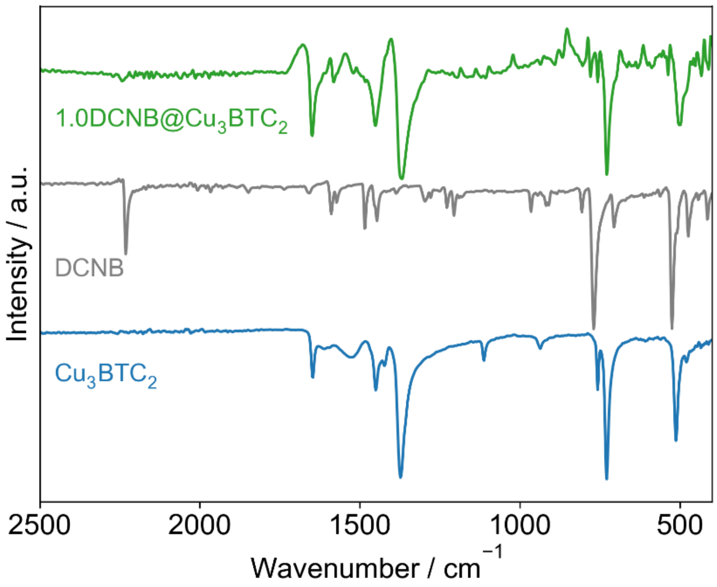

Supplementary Figure 25: IR transmission spectrum of pristine Cu_3BTC_2, DCNB and DCNB@Cu_3BTC_2. The spectra are background corrected and normalized. A vertical offset is applied for visualization.

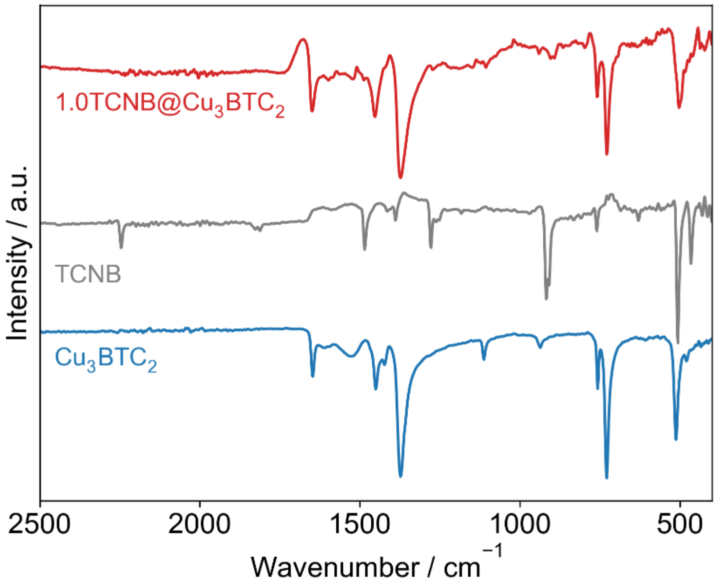

Supplementary Figure 26: FTIR transmission spectrum of pristine Cu_3BTC_2, TCNB and TCNB@Cu_3BTC_2. The spectra are background corrected and normalized. A vertical offset is applied for visualization.

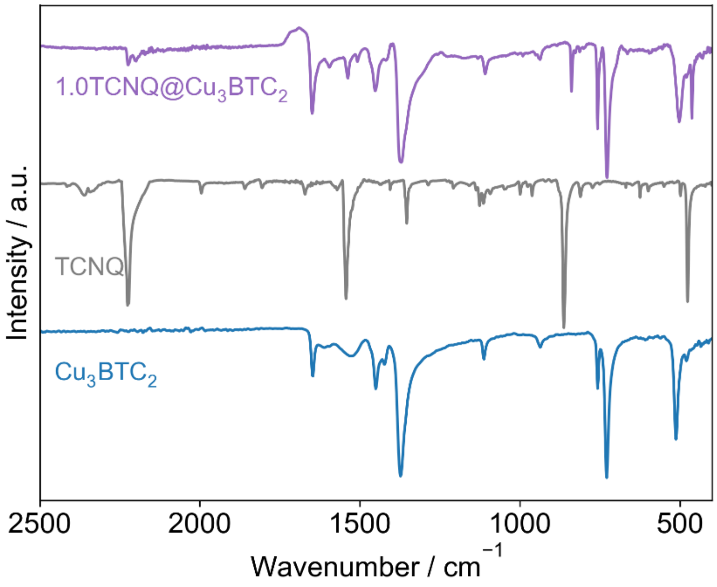

Supplementary Figure 27: FTR transmission spectrum of pristine Cu_3BTC_2, TCNQ and TCNQ@Cu_3BTC_2. The spectra are background corrected and normalized. A vertical offset is applied for visualization.

Supplementary Note 11: Scanning electron microscopy

SEM images were recorded using a JEOL JSM-7500F field emission scanning electron microscope operated in gentle beam mode.

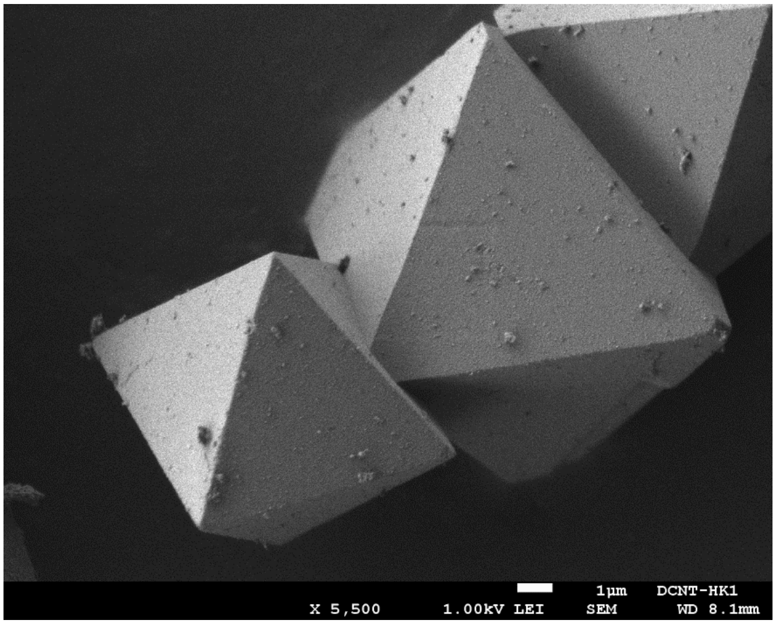

Supplementary Figure 28: SEM image of 1.0DCNT@Cu_3BTC_2.

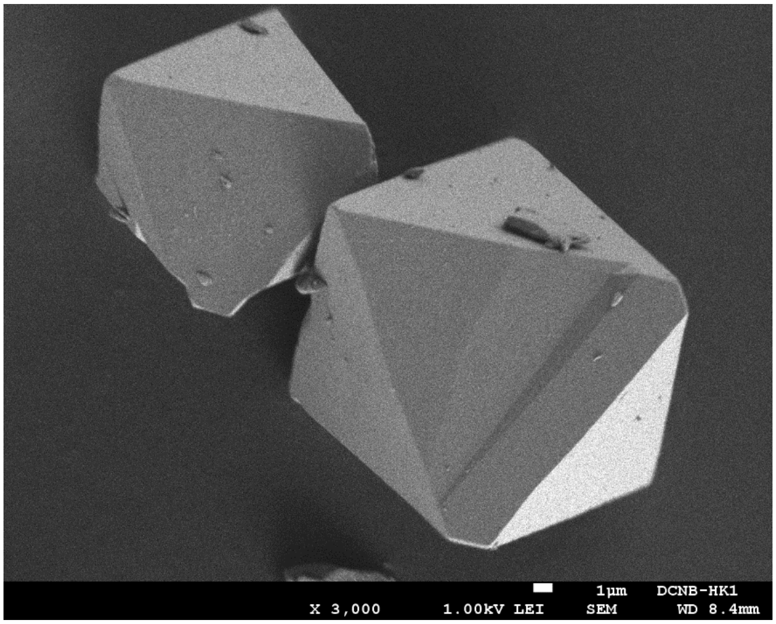

Supplementary Figure 29: SEM image of 1.0DCNB@Cu_3BTC_2.

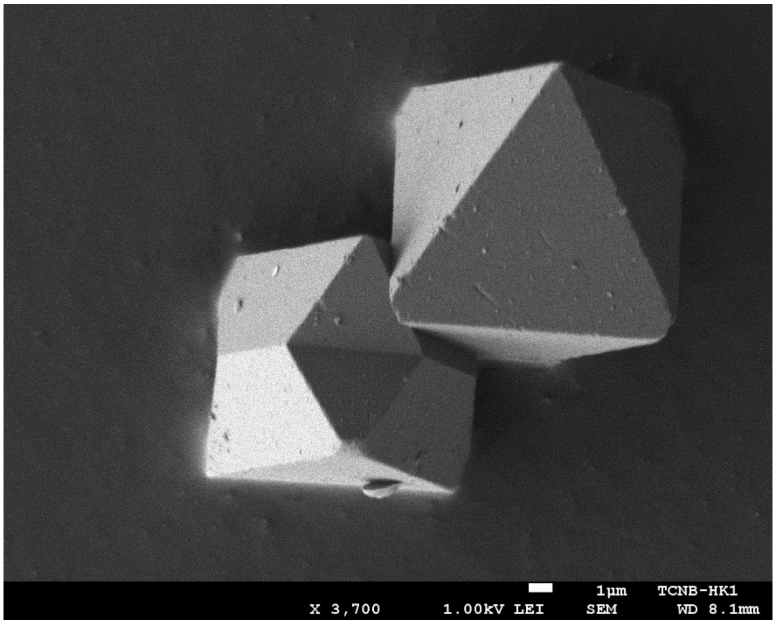

Supplementary Figure 30: SEM image of 1.0TCNB@Cu_3BTC_2.

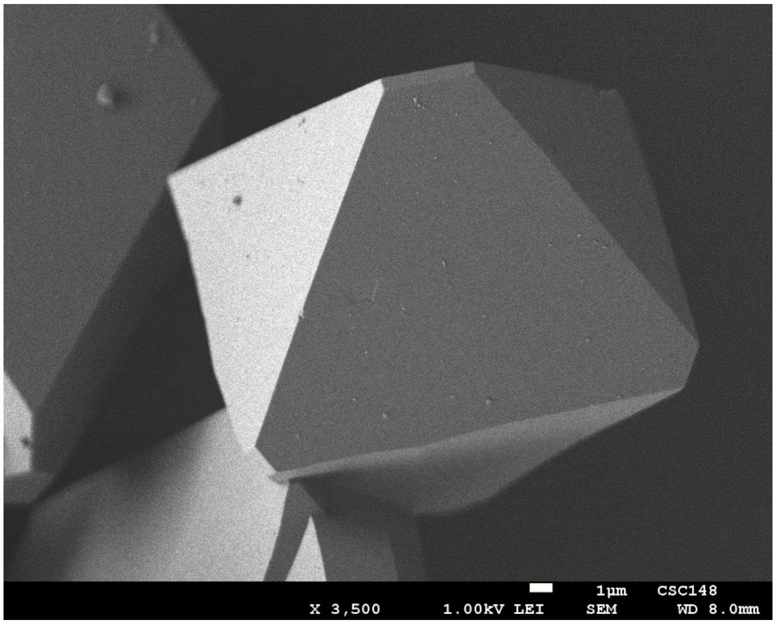

Supplementary Figure 31: SEM image of 1.75TCNE@Cu_3BTC_2.

Supplementary Note 12: Electrical conductivity measurements

The electrical conductivity of the materials was determined by *IV* measurements in a 2-point probe press cell. Inside the glovebox, powders were filled into the cell and compressed between stainless steel cylinders (1 cm diameter) at 3 t. A detailed description of the cell can be found elsewhere.[15] The *IV*-curves were recorded using a Gamry Reference 3000 potentiostat.

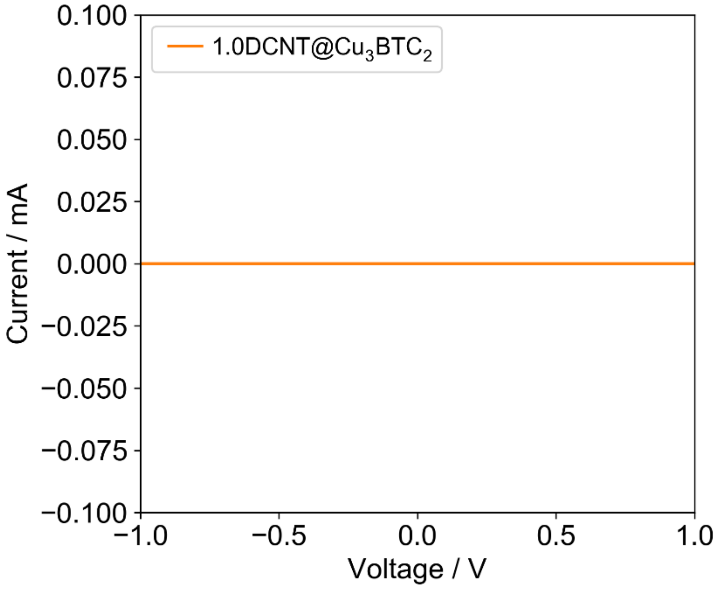

Supplementary Figure 32: *IV* curve of DCNT@Cu_3BTC_2.

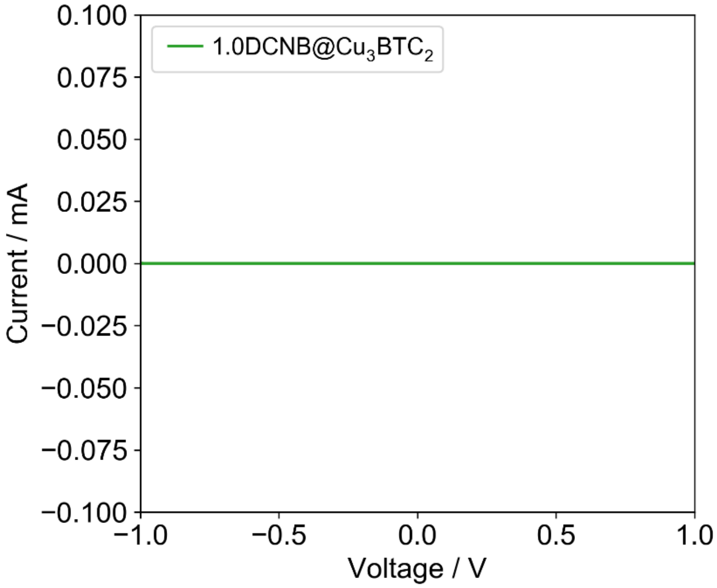

Supplementary Figure 33: *IV* curve of DCNB@Cu_3BTC_2.

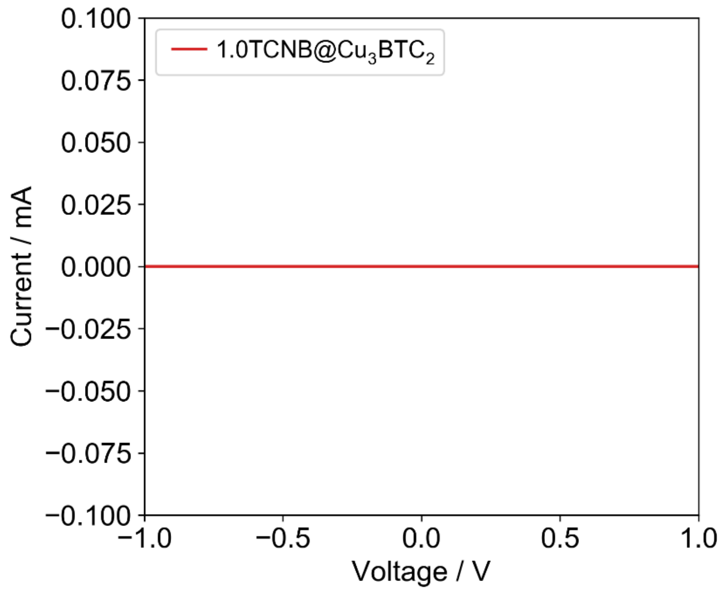

Supplementary Figure 34: *IV* curve of TCNB@Cu_3BTC_2.

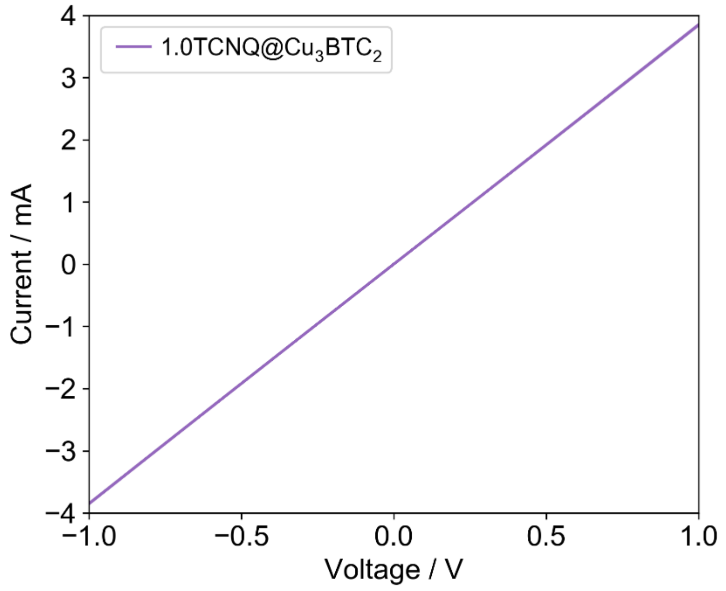

Supplementary Figure 35: *IV* curve of TCNQ@Cu_3BTC_2. The conductivity is in the order of 10^{-4} S/cm.

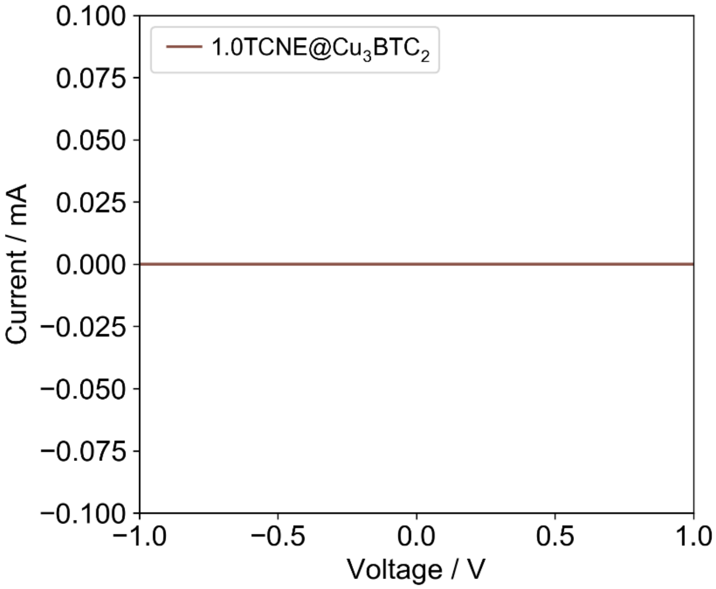

Supplementary Figure 36: *IV* curve of TCNE@Cu_3BTC_2.

We observed no current for any of the host-guest materials with DCNT, DCNB, TCNB, or TCNE as the guest. The insulating character of these materials can be the result of different redox potentials of the guests. For instance, the one electron reduction potential for TCNB is -0.71 V vs. S.C.E. and 0.19 V vs. S.C.E. for TCNQ.[16] However, the reduction potential of TCNE (0.24 V vs. S.C.E.[16]) is very close to the one of TCNQ, but we did not observe any conductivity for TCNE@Cu_3BTC_2 either. As we observe significant structural changes from the PXRD data of TCNE@Cu_3BTC_2 the interpretation of the properties is rather speculative. Under consideration of the SEM images, indicating no by-phase for any of the new host guest materials, it is possible that the CuTCNQ by-phase is the main contributor to the electrical conductivity of TCNQ@Cu_3BTC_2 prepared via vapor phase infiltration.

Supplementary References

1. Frisch M. J., et al. Gaussian 16 Rev. B.01. (ed^(eds) (2016).
2. Ditchfield R., Hehre W. J., Pople J. A. Self-Consistent Molecular-Orbital Methods. IX. An Extended Gaussian-Type Basis for Molecular-Orbital Studies of Organic Molecules. *J. Chem. Phys.* **54**, 724-728 (1971).
3. Lee C., Yang W., Parr R. G. Development of the Colle-Salvetti Correlation-Energy Formula into a Functional of the Electron Density. *Phys. Rev. B* **37**, 785-789 (1988).
4. Ahlrichs R., Bär M., Häser M., Horn H., Kölmel C. Electronic structure calculations on workstation computers: The program system turbomole. *Chem. Phys. Lett.* **162**, 165-169 (1989).
5. Becke A. D. A new Mixing of Hartree–Fock and Local Density-Functional Theories. *J. Chem. Phys.* **98**, 1372-1377 (1993).
6. Weigend F., Häser M., Patzelt H., Ahlrichs R. RI-MP2: Optimized Auxiliary Basis Sets and Demonstration of Efficiency. *Chem. Phys. Lett.* **294**, 143-152 (1998).
7. Treutler O., Ahlrichs R. Efficient molecular numerical integration schemes. *J. Chem. Phys.* **102**, 346-354 (1995).

8. Sierka M., Hogekamp A., Ahlrichs R. Fast evaluation of the Coulomb potential for electron densities using multipole accelerated resolution of identity approximation. *J. Chem. Phys.* **118**, 9136-9148 (2003).
9. Eichkorn K., Treutler O., Öhm H., Häser M., Ahlrichs R. Auxiliary basis sets to approximate Coulomb potentials. *Chem. Phys. Lett.* **240**, 283-290 (1995).
10. Eichkorn K., Weigend F., Treutler O., Ahlrichs R. Auxiliary basis sets for main row atoms and transition metals and their use to approximate Coulomb potentials. *Theor. Chem. Acc.* **97**, 119-124 (1997).
11. Grimme S., Antony J., Ehrlich S., Krieg H. A consistent and accurate ab initio parametrization of density functional dispersion correction (DFT-D) for the 94 elements H-Pu. *J. Chem. Phys.* **132**, 154104 (2010).
12. Elmali A. The Magnetic Super-Exchange Coupling in Copper(II) Acetate Monohydrate and a Redetermination of the Crystal Structure. *Turk. J. Phys.* **24**, 667-672 (2000).
13. Tafipolsky M., Amirjalayer S., Schmid R. First-Principles-Derived Force Field for Copper Paddle-Wheel-Based Metal−Organic Frameworks. *J. Phys. Chem. C* **114**, 14402-14409 (2010).
14. Talin A. A., et al. Tunable Electrical Conductivity in Metal-Organic Framework Thin-Film Devices. *Science* **343**, 66-69 (2014).
15. Schneider C., et al. High Electrical Conductivity and High Porosity in a Guest@MOF Material: Evidence of TCNQ Ordering within Cu3BTC2 Micropores. *Chem. Sci.* **9**, 7405-7412 (2018).
16. Peover M. E. Reduction potentials and intermolecular charge-transfer spectra of organic acceptor molecules. Part 4.-Nitrobenzenes. *J. Chem. Soc. Faraday Trans.* **60**, 479-483 (1964).
